# Glucocorticoids Improve Myogenic Differentiation In Vitro by Suppressing the Synthesis of Versican, a Transitional Matrix Protein Overexpressed in Dystrophic Skeletal Muscles

**DOI:** 10.3390/ijms18122629

**Published:** 2017-12-06

**Authors:** Natasha McRae, Leonard Forgan, Bryony McNeill, Alex Addinsall, Daniel McCulloch, Chris Van der Poel, Nicole Stupka

**Affiliations:** 1 School of Medicine, Deakin University, Waurn Ponds, VIC 3216, Australia; nmcrae@deakin.edu.au (N.M.); leonard.forgan@deakin.edu.au (L.F.); bryony.mcneill@deakin.edu.au (B.M.); aaddinsa@deakin.edu.au (A.A.); 2 Faculty of Law, The University of Queensland, Brisbane, QLD 4072, Australia; daniel.mcculloch@uq.net.au; 3 Department of Physiology, Anatomy and Microbiology, La Trobe University, Bundoora, VIC 3086, Australia; c.vanderpoel@latrobe.edu.au

**Keywords:** Duchenne muscular dystrophy, fibrosis, glucocorticoids, myogenesis, *mdx* mouse, versican

## Abstract

In Duchenne muscular dystrophy (DMD), a dysregulated extracellular matrix (ECM) directly exacerbates pathology. Glucocorticoids are beneficial therapeutics in DMD, and have pleiotropic effects on the composition and processing of ECM proteins in other biological contexts. The synthesis and remodelling of a transitional versican-rich matrix is necessary for myogenesis; whether glucocorticoids modulate this transitional matrix is not known. Here, versican expression and processing were examined in hindlimb and diaphragm muscles from *mdx* dystrophin-deficient mice and C57BL/10 wild type mice. V0/V1 versican (*Vcan*) mRNA transcripts and protein levels were upregulated in dystrophic compared to wild type muscles, especially in the more severely affected *mdx* diaphragm. Processed versican (versikine) was detected in wild type and dystrophic muscles, and immunoreactivity was highly associated with newly regenerated myofibres. Glucocorticoids enhanced C2C12 myoblast fusion by modulating the expression of genes regulating transitional matrix synthesis and processing. Specifically, *Tgfβ1, Vcan* and hyaluronan synthase-2 (*Has2*) mRNA transcripts were decreased by 50% and *Adamts1* mRNA transcripts were increased three-fold by glucocorticoid treatment. The addition of exogenous versican impaired myoblast fusion, whilst glucocorticoids alleviated this inhibition in fusion. In dystrophic *mdx* muscles, versican upregulation correlated with pathology. We propose that versican is a novel and relevant target gene in DMD, given its suppression by glucocorticoids and that in excess it impairs myoblast fusion, a process key for muscle regeneration.

## 1. Introduction

Duchenne muscular dystrophy (DMD) is a fatal hereditary disease affecting ~1:3500 boys, with glucocorticoid therapy being the only treatment with clinical efficacy [1]. DMD is caused by mutations in the dystrophin (*DMD*) gene, which renders dystrophic skeletal muscles vulnerable to ongoing contraction-induced injury, resulting in excessive inflammation, impaired regeneration and fibrosis [2]. Whilst fibrosis is usually thought of as a disease endpoint, it is important to note that endomysial extracellular matrix (ECM) accumulation precedes overt muscle degeneration in DMD [3], and is thought to actively contribute to the degeneration of dystrophic muscles [4,5,6].

The composition and processing of the ECM influences global cell behaviour, including cellular processes necessary for effective muscle repair [7,8,9]. Aberrant ECM synthesis and processing is observed in dystrophic muscles from patients with DMD [10] and in *mdx* mice [11], compromising regenerative myogenesis and exacerbating inflammatory processes [12]. TGF-β is considered to be a key cytokine driving fibrosis in DMD [13], and its levels are elevated in dystrophic muscles and in circulation [14].

The mature ECM of normal skeletal muscle is composed of glycoproteins, collagens and proteoglycans containing heparan sulphate and chondroitin sulphate/dermatan sulphate glycosaminoglycan (GAG) side chains [15]. Endomysial fibrosis in DMD is associated with the increased expression of not only mature ECM proteins [16,17], but also transitional ECM proteins such as hyaluronan and versican [18]. These transitional matrix proteins, through their synthesis and remodelling, regulate cell behaviour during normal development and regeneration, as well as functioning as a scaffold for mature ECM deposition [8,19]. ECM proteases are also upregulated in dystrophic muscles [20]. Strategies to limit aberrant ECM synthesis and remodelling in DMD are of therapeutic interest. Given the importance of a transitional matrix in tissue repair, versican is an especially relevant ECM protein [18].

Versican is a chondroitin sulphate proteoglycan (CSPG) [19], localised to pericellular regions of the basement membranes and interstitial matrices [9,21,22]. In skeletal muscle, the V0 and V1 splice variants of versican are the most abundant [9]. V0/V1 versican is comprised of the G1 and G3 globular domains at the N- and C-terminus respectively, with each of their core proteins sharing a common GAG-β domain and V0 versican containing an additional GAG-α domain. Chondroitin sulphate moieties on V0/V1 versican bind growth factors, cytokines and adhesion molecules, such as CD44 (cluster of differentiation 44), to regulate downstream signalling pathways [23]. The C- and N-termini of versican bind various ECM molecules [24], including hyaluronan [25], a large non-proteinaceous GAG of variable size which has been linked to myogenesis and muscle growth [8,26]. Hyaluronan is synthesised by hyaluronan synthases (HAS) and degraded by hyaluronidases (HYAL), with HAS2, HYAL1 and HYAL2 being the predominant skeletal muscle isoforms [26].

Recent studies have highlighted the role of versican in myoblast proliferation and myotube formation, processes critical for regenerative myogenesis [9,27]. In developing chick skeletal muscle, versican is synthesised early in myogenesis [27] and is localised to the pericellular matrix of developing myotubes [28]. V1 versican is cleaved at the Glu^441^-Ala^442^ peptide bond within the GAG-β domain by specific A Disintegrin-like And Metalloproteinase Domain with ThromboSpondin-1 repeats (ADAMTS) proteoglycanases [29], which include ADAMTS1, -4, -5, -9, -15 and -20 [30], and presumably ADAMTS8, however this has not yet been proven [31]. This produces the bioactive G1-DPEAAE fragment known as versikine [19], which in other biological contexts can be pro-apoptotic [32] or pro-inflammatory [33]. Using C2C12 myoblasts as an in vitro model of regenerative myogenesis, we have shown that the processing of a versican and hyaluronan rich transitional, pericellular matrix by ADAMTS5 or -15 facilitated myotube formation [9].

Glucocorticoids delay disease progression in DMD by improving muscle strength, respiratory function, and increasing ambulation by up to four years [34,35]. These beneficial effects may be due to membrane stabilisation, decreased muscle necrosis and fibrosis, modulation of inflammation, and/or improved regeneration [12,36]. In dystrophic *mdx* mice, high dose treatment with the glucocorticoid deflazacort increased the proliferation and/or fusion of muscle precursor cells during myotube formation following crush injury, as well as enhancing the growth of intact myotubes [37]. More recently, when *mdx* mice were treated with prednisone or VBP15 (vamorolone; a dissociative glucocorticoid [38]) TGF-β related networks were suppressed, this included reduced gene expression of various collagen isoforms (1A1, 3A1 and 6A1), leading to improved muscle repair [12]. In vitro, glucocorticoids also enhanced myotube formation in primary wild type and dystrophic *mdx* myoblasts, as well as in C2C12 cells [39,40]. 

Given the importance of transitional matrix synthesis and remodelling for myofibre formation and that glucocorticoids can enhance myogenic differentiation [1,8,41], the effects of glucocorticoids on the ECM during myogensis and myoblast differentiation should be better characterised. In other disease models associated with a heightened pro-inflammatory state, glucocorticoids do modulate ECM composition and degradation with the specific effects being tissue and context dependent. In cultured rat mesangial cells, glucocorticoids decreased secreted and cell associated chondroitin sulphate and dermatan sulphate proteoglycan content by 50% [42]. In cultured airway fibroblasts stimulated with serum, glucocorticoids decreased versican gene and protein expression by 50% [43]. Hyaluronan content in skin and dermal fibroblasts was also reduced following glucocorticoid treatment [44], due to decreased *Has2* mRNA transcription and stability, and thus reduced hyaluronan synthesis [45]. Given their clinical usage in DMD and the ECM expansion observed in dystrophic muscles, understanding the transitional ECM gene targets regulated by glucocorticoids in skeletal muscle cells is an important step towards improving therapeutic outcomes for strategies targeting fibrosis in dystrophy.

Here, we show that V0/V1 versican mRNA transcript abundance and protein levels are elevated in diaphragm and hindlimb tibialis anterior (TA) muscles from dystrophic *mdx* mice when compared to C57BL/10 wild type mice. We identify a novel mechanism mediating the glucocorticoid stimulated increase in myoblast fusion and myotube formation in C2C12 cells. Specifically, in differentiating myoblasts, low doses of glucocorticoids (25 or 100 nM dexamethasone) modulated the expression of genes associated with the synthesis and processing of a versican–hyaluronan rich transitional matrix. This effect is dependent on versican, as glucocorticoid treatment improved myotube formation in the presence of excess versican. As a whole, these findings offer novel insight into the relevance of versican in dystrophic skeletal muscle pathology. 

## 2. Results

### 2.1. Versican Expression Is Increased in Dystrophic Muscles and Correlate with the Severity of Pathology

**Figure 1:**
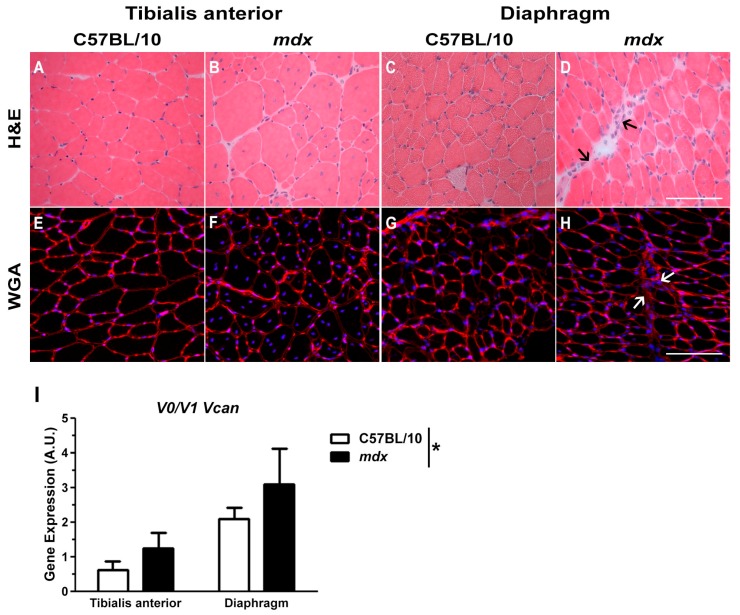
Muscle architecture and fibrosis in wild type and *mdx* mice. (**A**–**D**) Muscle architecture can be determined from the H and E-stained muscle cross-sections, and (**E**–**H**) areas of fibrosis can be observed by examining wheat germ agglutinin (WGA) stained cross-sections. (**I**) Versican (*V0/V1 Vcan*) mRNA transcript abundance was increased in *mdx* compared to wild type mice (* *p* = 0.026; main effect genotype; 2-way general linear model (GLM) ANOVA). Arrows denote areas of fibrosis and mononuclear infiltrate. Gene expression analysis was determined from *n* = 3 wild type mice and *n* = 3 *mdx* mice. Scale bar = 100 µm. Error bars = S.E.

**Figure 2:**
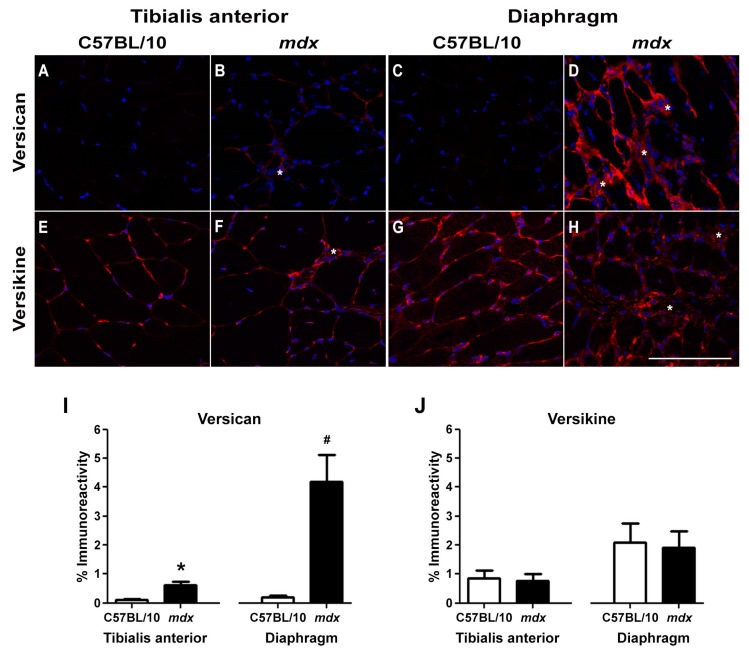
Versican and versikine expression in TA and diaphragm muscles from wild type and *mdx* mice. Representative images of (**A**–**D**) versican and (**E**–**H**) versikine immunoreactivity from TA and diaphragm muscles from wild type and *mdx* mice. (**I**) Quantification of versican immunoreactivity revealed an upregulation in TA (* *p* = 0.001) and diaphragm muscles (^#^ *p* = 0.0001) from *mdx* mice when compared to wild type mice. (**J**) Versikine immunoreactivity was similar in *mdx* and wild type TA or diaphragm muscles. White asterisks denote areas of mononuclear infiltration. Immunoreactivity analysis was determined from *n* = 5 wild type mice and *n* = 5 *mdx* mice. Scale bar = 100 µm. Error bars = S.E.

The pathology of TA muscles from adult *mdx* mice is moderate compared to the human disease. Specifically, in hindlimb muscles from *mdx* mice there is minimal fibrosis, lower levels of inflammation and more effective regeneration as indicated by the presence of centrally nucleated fibres [46] (Figure 1B); although, muscle strength is compromised [47]. The pathology of *mdx* diaphragm muscles is more representative of DMD, with greatly impaired contractile function and high levels of endomysial fibrosis [48,49] (Figure 1D). *V0/V1 Vcan* mRNA transcript abundance was greater in dystrophic compared to wild type muscles (Figure 1I). To support this gene expression data, the immunoreactivity of full length V0/V1 versican and its cleaved bioactive fragment, versikine, was assessed in TA and diaphragm muscle cross-sections from *mdx* and wild type mice (Figure 2).

**Figure 3:**
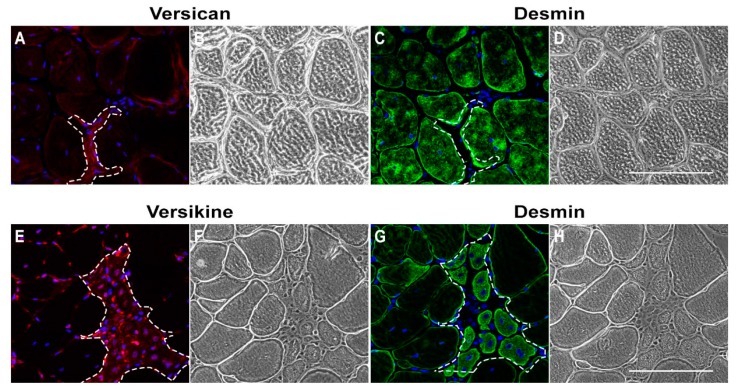

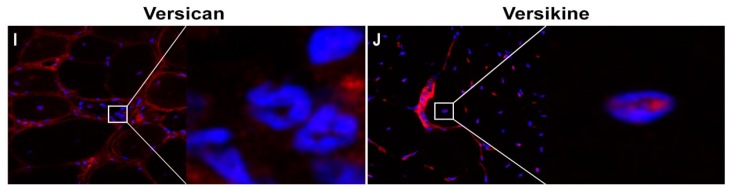
Versikine is localised to regenerating myofibres and mononuclear infiltrate, as well as within nuclei in *mdx* TA muscles. Serial cross-sections were stained with versican or versikine and desmin, phase images were captured to confirm localisation and tissue orientation. (**A**–**D**) Versican was localised to interstitium between myofibres. (**E**–**H**) Versikine was highly expressed in regenerating muscle, as indicated by its association with small desmin positive, centrally nucleated myofibres. (**I**,**J**) In dystrophic muscle cross-sections, nuclear localisation of versikine, but not versican, was also observed. Scale bars = 100 µm.

V0/V1 versican protein levels were upregulated in TA and diaphragm muscles from *mdx* mice (Figure 2B,D) compared to wild type mice (Figure 2A,C, and as quantified in Figure 2I). In concordance with the more severe pathology, versican immunoreactivity was greatest in the *mdx* diaphragm. In dystrophic muscles, versican staining was localised to regions of mononuclear infiltrate, which includes myoblasts, inflammatory cells and fibroblasts (Figure 2B,D; as indicated with a white asterisk). Versican staining was also associated with endomysial fibrosis in TA (Figure 3A–D) and diaphragm muscles (Figure 2D).

Remodelling of V1 versican by ADAMTS proteoglycanases (e.g., ADAMTS1, -5 and -15) yields the bioactive versikine fragment [19]. Versikine immunoreactivity was predominantly localised to the pericellular region of myofibres, and did not differ between dystrophic and wild type TA or diaphragm muscles (Figure 2E–H, and as quantified in Figure 2J). In *mdx* TA and diaphragm muscle cross-sections, versikine staining was also associated with regions of mononuclear infiltrate (Figure 2F,H; as indicated with a white asterisk). When this association of versikine with mononuclear infiltrate was further interrogated in *mdx* TA muscle cross-sections by specifically assessing areas of regeneration, high levels of versikine immunoreactivity were co-localised with desmin positive, newly regenerated myofibres (Figure 3E–H). Furthermore, nuclear localisation of versikine (but not versican) was observed in muscle fibres and mononuclear infiltrate (Figure 3I,J).

### 2.2. Glucocorticoids Enhance Myoblast Fusion and Myotube Formation

**Figure 4:**
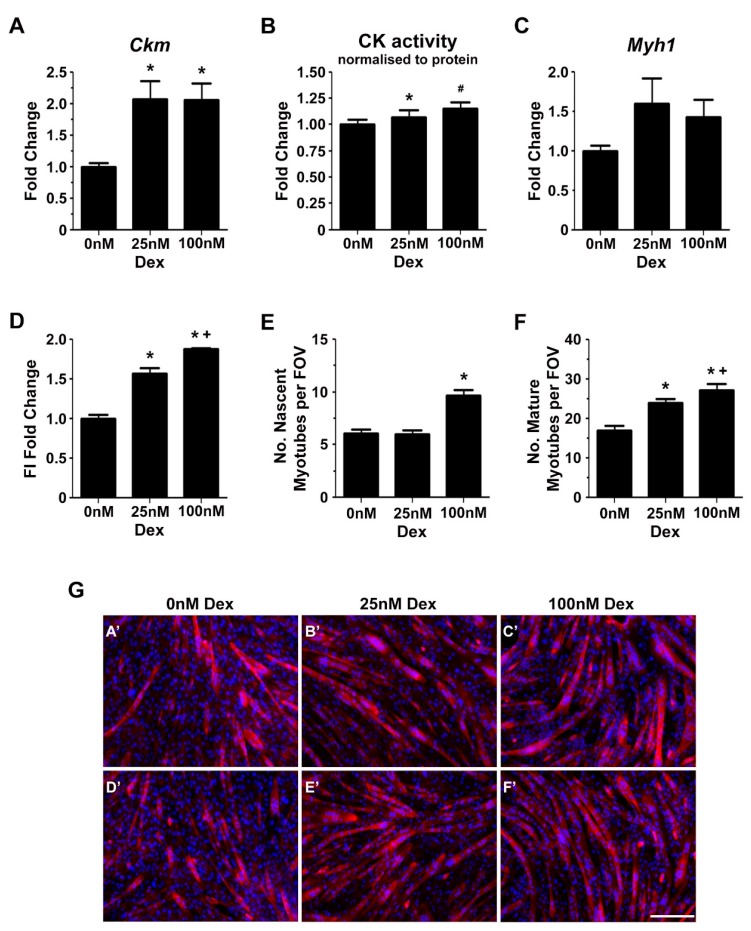
Low dose dexamethasone treatment for 72 h increased myogenic differentiation efficacy in C2C12 myoblasts. (**A**) The gene expression of myogenic differentiation marker creatine kinase (*Ckm*) was increased two-fold in cells treated with 25 nM and 100 nM dexamethasone (Dex) (* *p* < 0.01); (**B**) CK enzyme activity in cell lysates was also increased following treatment with 25 nM (* *p* = 0.02) and 100 nM dexamethasone (^#^ *p* = 0.01); (**C**) Dexamethasone (Dex) did not significantly increase myosin heavy chain 1 (*Myh1*) mRNA transcripts; (**D**) Fusion index (FI; * *p <* 0.001) was greater in C2C12 cells treated with 25 nM and 100 nM dexamethasone compared to untreated control cells, and this increase was dose dependent (^+^ *p* < 0.05); (**E**) Treatment with 100 nM dexamethasone increased the formation of nascent myotubes containing 3–4 myonuclei (* *p* < 0.001); (**F**) The number of mature myotubes with ≥5 nuclei was greater in cultures treated with 25 nM or 100 nM versus 0 nM dexamethasone (* *p* = 0.017), and this increase was dose dependent (^+^ *p* = 0.03); (**G**) Representative images of C2C12 myotubes. Differentiation C2C12 myoblasts were treated with 0 nM dex (**A’**,**D’**), 25nM dex (**B’**,**E’**) or 100 nM dex (**C’**,**F’**) for 72 h. Myotubes were then stained with phalloidin for F-actin (red) and DAPI for nuclei (blue). CK enzyme activity was calculated from *n* = 3 biological replicates performed in quadruplicate. The fusion index and myotube number were calculated from *n* = 3 biological replicates performed in duplicate. Gene expression was determined from *n* = 3 biological replicates in triplicate. Scale bar = 200 µm. Error bars = S.E.

C2C12 myoblasts were used to investigate glucocorticoid mediated effects on transitional matrix synthesis and remodelling during myogenic differentiation. Similar to skeletal muscle development in vivo, the myogenic differentiation of C2C12 myoblasts is associated with an upregulation of transitional matrix genes, such as *V1/V0 Vcan*, *Adamts1*, *Adamts5*, *Adamts15*, *Pcsk6* [9], *Has2* and *Hyal2* [8,41]. The two-fold increase in the gene expression of the myogenic differentiation marker creatine kinase muscle (*Ckm*) (Figure 4A) and the associated dose dependent increase in total creatine kinase (CK) enzyme activity (Figure 4B), indicate that low dose glucocorticoid treatment enhanced myogenic differentiation. Essential to myogenic differentiation is the fusion of myoblasts into multinucleated myotubes, a multistep process involving migration, alignment, adhesion and membrane coalescence to form nascent myotubes [50]. Subsequent growth of these nascent myotubes occurs through the incorporation of additional myoblasts via secondary fusion [51,52]. In accordance with previously published findings [39,40], the fusion index, which is the proportion of nuclei fused into multinucleated myotubes compared to total nuclei, was increased in a dose dependent manner following treatment with 25 nM and 100 nM dexamethasone (Figure 4D). Myotube formation and maturation is considered to be a two-step process, with distinct signaling pathways contributing to the formation of nascent myotubes and the growth of mature myotubes [53,54,55]. Here, nascent myotubes containing 3–4 myonuclei and growing, mature myotubes undergoing secondary fusion and containing ≥5 myonuclei, were quantified as previously described [9]. In concordance with the fusion index data, there was an increase in the number of nascent myotubes (with 3–4 myonuclei) following treatment with 100 nM dexamethasone (Figure 4E), as well as a dose dependent increase in the number of mature myotubes with ≥5 myonuclei indicating enhanced secondary myoblast fusion (Figure 4F).

### 2.3. Glucocorticoids Regulate the Expression of Genes Associated with Transitional Matrix Synthesis and Processing during Myogenic Differentiation

**Figure 5:**
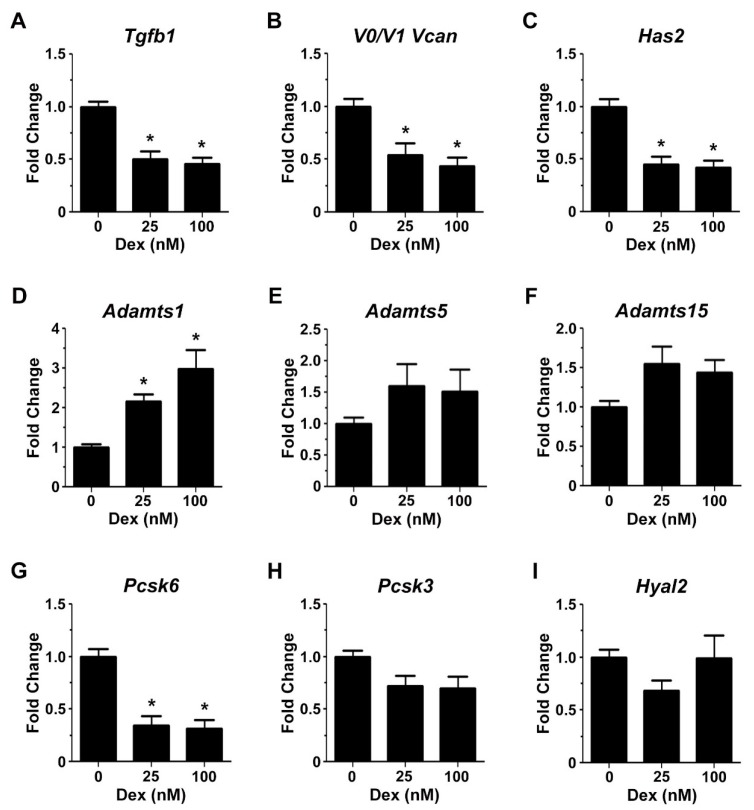
The expression of genes associated with a versican–hyaluronan rich transitional matrix is modulated by dexamethasone during myoblast differentiation. (**A**–**C**) Compared to untreated control cells, 25 nM or 100 nM dexamethasone treatment decreased *Tgfb1* (* *p* < 0.0001 and * *p* = 0.0001 respectively), *Vcan* (* *p* = 0.005 and * *p* = 0.0006, respectively) and *Has2* mRNA transcripts by approximately two-fold (* *p* < 0.0001 and * *p* = 0.0001, respectively); (**D**) *Adamts1* gene expression was increased up to three-fold in response to 25 nM and 100 nM dexamethasone treatment (* *p* = 0.03 and * *p* < 0.001, respectively); (**E**,**F**) *Adamts5* and *Adamts15* mRNA transcripts were not significantly increased; (**G**,**H**) *Pcsk6*, but not *Pcsk3*, mRNA transcripts were decreased approximately two-fold following treatment with 25 and 100 nM dexamethasone (* *p* < 0.0001). (**I**) *Hyal2* mRNA levels were not altered by dexamethasone treatment. Gene expression was determined from *n* = 3 biological replicates in triplicate. Error bars = S.E.

Glucocorticoids may enhance myogenic differentiation by regulating the expression of genes associated with transitional matrix synthesis and processing. Specifically, 72 h of treatment with 25 nM and 100 nM of dexamethasone reduced *Tgfb1* mRNA transcript abundance by 50% and 54%, respectively (Figure 5A). The decrease in *Tgfb1* mRNA transcript abundance was associated with a reduction in versican (*V0/V1 Vcan*) gene expression by up to 56% (Figure 5B) and *Has2* gene expression by up to 58% (Figure 5C). *Has2* is the primary *Has* gene involved in hyaluronan synthesis in skeletal muscle [26]. *Adamts1* mRNA transcripts were increased up to three-fold in dexamethasone treated C2C12 cells (Figure 5D), whereas *Adamts5* or *Adamts15* mRNA levels were not affected by glucocorticoid treatment (Figure 5E,F). ADAMTS proteoglycanases are synthesised as inactive zymogens and become activated upon proteolytic processing by Furin and/or Pace4 [56]. Treatment with 25 and 100 nM dexamethasone decreased *Pcsk6* (Pace4) gene expression by 65% and 68% respectively (Figure 5G), whilst *Pcsk3* (Furin) mRNA levels were not significantly altered (Figure 5H). Lastly, dexamethasone had no effect on *Hyal2* mRNA transcript abundance (Figure 5I), the enzyme necessary for hyaluronan degradation [26]. 

**Figure 6:**
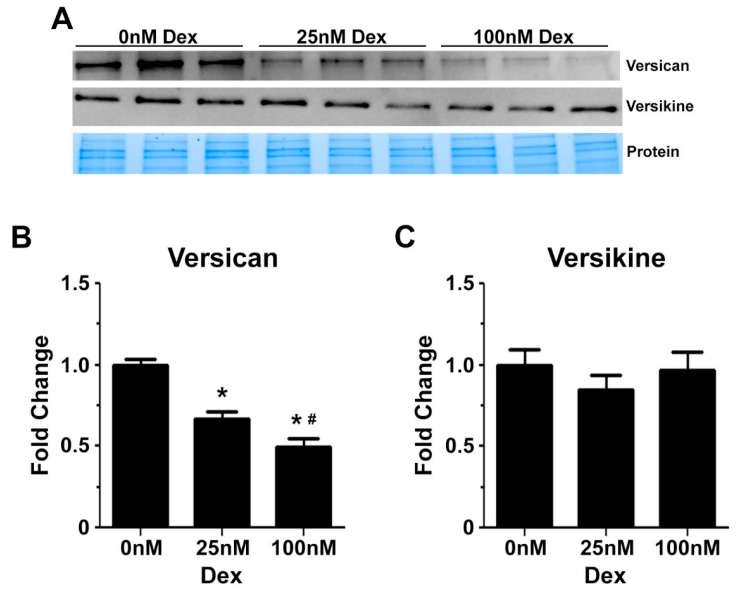
Versican and versikine protein expression in differentiating C2C12 myoblasts following dexamethasone treatment. (**A**) Representative western blots to assess versican and versikine expression in C2C12 cell lysates, with the respective stain free protein gel image to demonstrate even protein loading; (**B**) Decreased versican protein expression following treatment with 25 nM (* *p* = 0.00002) and 100 nM dexamethasone (* *p* = 0.0000001); and this decrease was dose dependent (^#^ *p* = 0.03); (**C**) Versikine protein levels were not altered by dexamethasone treatment. Versican and versikine protein expression analysis was calculated from *n* = 3 biological replicates performed in quadruplicate. Error bars = S.E.

The effect of glucocorticoids on versican gene expression was confirmed by western blotting. Dexamethasone reduced protein levels of full length V0/V1 versican in a dose dependent manner by up to 50% (Figure 6B). ADAMTS dependent remodelling of versican during myogenic differentiation appeared not to be altered by glucocorticoids, as indicated by similar protein levels of versikine following treatment with 25 nM and 100 nM dexamethasone (Figure 6C).

### 2.4. Glucocorticoids Rescue Myotube Formation in Differentiating Myoblasts Treated with Exogenous Versican and Versikine

**Figure 7:**
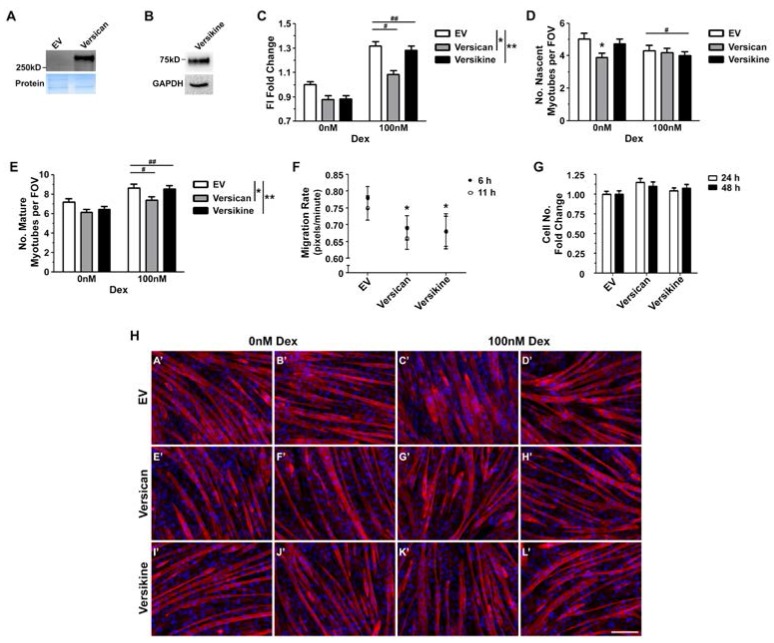
Dexamethasone ameliorated the impairment in myogenic differentiation associated with excess versican. (**A**,**B**) Representative western blots of versican, versikine or empty vector (EV) conditioned media, with the respective images of stain free protein gels or GAPDH as a loading controls; (**C**) The addition of versican and versikine conditioned media compromised myoblast differentiation, as assessed by fusion index (main effect conditioned media; 2-way GLM ANOVA; * *p* < 0.001 for versican conditioned media and ** *p* < 0.02 for versikine conditioned media relative to cells treated with the empty vector conditioned media). Whilst, glucocorticoids enhanced myoblast fusion (main effect dexamethasone; 2-way GLM ANOVA; ^#^ *p <* 0.001 for empty vector or versican treated cells and ^##^ *p* < 0.001 empty vector or versikine treated cells); (**D**) In the absence of dexamethasone, excess versican reduced the formation of nascent myotubes (interaction; 2-way GLM ANOVA; * *p <* 0.001). In response to 100 nM dexamethasone, the number of nascent myotubes was similar in cells treated with versican or empty vector conditioned media. Versikine had no effect on nascent myotube formation, whilst dexamethasone decreased nascent myotube number in cells treated with versikine or empty vector conditioned media (main effect dexamethasone; 2-way GLM ANOVA; ^#^ *p <* 0.01); (**E**) Versican or versikine reduced the number of mature myotube formed per field of view (FOV) (main effect conditioned media; 2-way GLM ANOVA; * *p <* 0.0001 for versican treated cells and ** *p <* 0.0001 versikine treated cells), and 100 nM dexamethasone ameliorated this decrease in myotube number (main effect dexamethasone; 2-way GLM ANOVA; ^#^ *p* = 0.0092 for empty vector or versican treated cells and ^##^ *p* = 0.0061 empty vector or versikine treated cells); (**F**) Versican or versikine conditioned media reduced the migration rate of C2C12 myoblasts compared to empty vector conditioned media (* *p* = 0.01 and * *p* = 0.04 respectively); (**G**) Myoblast cell number was not different following 24 h and 48 h of treatment with versican or versikine conditioned media compared to empty vector conditioned media; (**H**) Representative images of C2C12 myotubes. Differentiating C2C12 myoblasts were treated with 0 nM dex and EV conditioned media (**A’**,**B’**), 100 nM dex and EV conditioned media (**C’**,**D’**), 0 nM dex and versican conditioned media (**E’**,**F’**), 100 nM dex and versican conditioned media (**G’**,**H’**), 0 nM dex and versikine conditioned media (**I’**,**J’**), or 100 nM dex and versikine conditioned media (**K’**,**L’**) for 72 h. Myotubes were then stained with phalloidin for F-actin (red) and DAPI for nuclei (blue). Fusion index (FI) and myotube number were calculated from *n* = 5 biological replicates performed in duplicate. Migration rate was measured from *n* = 5 biological replicates performed in duplicate or triplicate. Myoblast proliferation was assessed from *n* = 3 biological replicates performed in 8 wells. Scale bar = 200 µm. Error bars = S.E.

In vitro, versican processing facilitates myoblast fusion and myotube formation, whilst an excess of versican appears to be detrimental [9]. Therefore, it is possible that reduced versican synthesis may contribute to the positive effects of glucocorticoids on regenerative myogenesis in dystrophic muscles. To test this hypothesis, differentiating C2C12 myoblasts were treated with V1 versican, versikine or empty vector conditioned media supplemented with 0 nM or 100 nM dexamethasone. The addition of conditioned media made the experimental conditions more challenging, with greater variability in fusion between biological replicates and a blunted response to dexamethasone. Nonetheless, excess full length or processed versican decreased myoblast fusion (main effect conditioned media; 2-way GLM ANOVA; * *p <* 0.001 for versican treated cells and ** *p <* 0.02 for versikine treated cells; Figure 7C). This decrease in fusion was ameliorated with glucocorticoid treatment (main effect dexamethasone; 2-way GLM ANOVA; # *p <* 0.001 for empty vector or versican treated cells and ^##^ *p <* 0.001 for empty vector or versikine treated cells; Figure 7C).

As further evidence that versican impairs myoblast fusion, in cells treated with 0 nM dexamethasone, the versican conditioned media decreased the number of nascent myotubes compared to empty vector conditioned media (interaction; 2-way GLM ANOVA; * *p <* 0.001). Following treatment with 100 nM dexamethasone, the number of nascent myotubes was similar in cells treated with the versican or empty vector conditioned media (Figure 7D). Versikine had no effect on the number of nascent myotubes formed (Figure 7D). Unexpectedly, dexamethasone decreased the number of nascent myotube in cells treated with versikine or empty vector conditioned media (main effect dexamethasone; 2-way GLM ANOVA; ^#^ *p <* 0.01) (Figure 7D).

With regards to the effects of versican, versikine and glucocorticoids on secondary fusion, the number of mature myotubes was reduced in cells treated with versican or versikine conditioned media (main effect conditioned media; 2-way GLM ANOVA; * *p <* 0.0001 for versican treated cells and ** *p <* 0.0001 versikine treated cells; Figure 7E). When differentiating myoblasts were treated with 100 nM dexamethasone, the number of mature myotubes increased (main effect dexamethasone; 2-way GLM ANOVA; ^#^ *p =* 0.0092 for versican treated cells and ^##^ *p =* 0.0061 versikine treated cells; Figure 7E).

Alignment of myoblasts is essential for fusion, and this depends on carefully regulated migration [50,57,58]. Versican is known to modulate cell migration and depending on the biological context the effects can be stimulatory [59,60] or inhibitory [61]. The effects of versikine on cell migration have not been well characterised. In C2C12 cells treated with versican or versikine conditioned media for up to 11 h, myoblast migration rate was reduced by 12% and 13%, respectively (Figure 7F). Thus, excess versican, both the full-length protein and the cleaved bioactive fragment, may also impair regenerative myogenesis through a reduction in myoblast migration.

Myoblast viability and number can be a confounding factor in determining the efficacy of myogenic differentiation. Versican has been shown to increase proliferation in various biological contexts [62,63], including primary turkey myoblasts [27]. In contrast, versikine has been associated with apoptosis during interdigital web regression [32]. In actively proliferating C2C12 myoblast cultures, exogenous versican or versikine had no effect on cell number (Figure 6G). 

## 3. Discussion

In dystrophic skeletal muscles, excess synthesis and inappropriate processing of ECM proteins lead to degeneration, fibrosis and compromised contractile function [17,64]. Similarities in the mechanisms of ECM expansion in patients with DMD and *mdx* mice have been observed, and contribute to the dystrophic pathology of these muscles [12]. The significance of versican in the generation and remodelling of a transitional matrix during skeletal muscle development and regeneration is continuing to gain recognition [8,9,27]. We propose that the carefully regulated synthesis and processing of a versican rich transitional matrix is also an important factor in differentiating between successful regenerative myogenesis or degeneration and fibrosis. A better understanding of versican function in muscular dystrophy is needed if progress is to be made in targeting the dysregulated ECM, which is a hallmark of DMD pathology.

Here, we report that the expression of full-length versican is increased in dystrophic *mdx* diaphragm and hindlimb muscles compared to wild type muscles, with the highest level of versican expression observed in the more severely affected *mdx* diaphragm muscles. These observations are in concordance with human data showing increased versican expression in muscle biopsies from patients with DMD compared to healthy controls, as assessed by immunohistochemistry [65] and microarray gene expression analysis [66]. Furthermore, deposition of chondroitin sulphate GAG side chains is upregulated in DMD [15], and V0/V1 versican is a significant source of chondroitin sulphate GAG chains in skeletal muscle. V0 versican is the most highly glycosylated isoform, followed by the V1 variant [67]. Versican is secreted and synthesised by activated satellite cells and myoblasts [27,68], newly formed myotubes [28], inflammatory cells [69] and fibroblasts [70].

Versican is transiently upregulated in myoblasts and newly formed myotubes during development and regeneration [28,71], whilst in healthy, mature skeletal muscle full length versican expression is quite low. Versican remodelling has been implicated in various developmental processes, and remodelling by specific ADAMTS proteoglycanases generates versikine [19,32]. Interestingly, in *mdx* TA muscles, versikine immunoreactivity was associated with small, recently regenerated myofibres. Furthermore, in dystrophic muscles, the nuclear localisation of versikine was observed in both muscle fibres and in mononuclear infiltrate. This is in line with observations by Carthy et al., who using the same anti-DPEAAE neo-epitope antibody (ThermoFisher Scientific, PA1-1748A, Waltham, MA, USA) detected nuclear versikine staining in vascular smooth muscle cells and proposed a potential role in mitotic spindle organization during cell division [63]. Versikine is further degraded by various ECM proteases. This hypothesis is supported by the observation that versikine immunoreactivity in developing mouse hindlimb muscles at E13.5 days is much higher than in mature muscles at 3 weeks of age [9]. This further degradation of versikine may account for the lack of difference in protein levels between diaphragm and TA muscles from *mdx* and wild type mice, despite increased V0/V1 versican expression.

In vivo, centrally nucleated fibres are indicative of recent damage and repair, with myoblast fusion being essential for effective regeneration. In diaphragm muscles from *mdx* mice, the proportion of centrally nucleated fibres is much lower, up to 2–3 folds, when compared to dystrophic TA muscles [48,72]. We hypothesise that excess versican accumulation contributes to the impaired regenerative capacity of *mdx* diaphragm muscles. As such, we propose that versican reduction could be a potential strategy to ameliorate the pathology of dystrophic muscles. In vitro evidence that processing of versican by ADAMTS5 or -15 facilitates myoblast fusion supports this hypothesis [9]. Furthermore, others have shown that the formation of multinucleated myotubes is associated with a reduction in chondroitin sulphate GAG sidechains, which also suggests a potential role for versican processing [73]. It is worth noting that versikine does not contain chondroitin sulphate GAG side chains [74].

Glucocorticoids improve muscle function in patients with DMD through various cellular mechanisms [36,75,76]. Of particular interest, are the effects of glucocorticoids on ECM synthesis and remodelling [77], on TGF-β [78] and TGF-β centred signalling networks, as these are highly relevant to regenerative myogenesis and fibrosis [12]. Our observation of a concentration dependent increase in myoblast fusion and myotube formation following low dose, 25 nM and 100 nM, dexamethasone treatment is in concordance with a number of studies reporting positive effects of glucocorticoids on myogenesis in vitro [39,40] and muscle regeneration in vivo [12,37]. In contrast to our findings, Ma et al. [79] reported inhibition of myogenic differentiation of C2C12 and primary mouse myoblasts following glucocorticoid treatment through the activation of glycogen synthase kinase 3β (GSK-3β). However, the concentration of dexamethasone used (10 μM) was 100–400 folds higher than our low dose treatment [79]. Furthermore, with lower concentrations of 10 nM and 100 nM dexamethasone, no significant decrease in myogenin or myosin heavy chain protein expression was reported, whilst the fusion index was not assessed [79].

The increase in myotube formation following glucocorticoid treatment was associated with decreased V0/V1 versican protein and *V0/V1 Vcan*, *Has2* and *Tgfb1* mRNA transcript abundance. This reduction in *Tgfb1* gene expression in response to glucocorticoids is in agreement with in vivo studies in *mdx* mice [12,78], and in vitro studies using hepatic stellate cells [80] and fetal lung fibroblasts [81]. In differentiating myoblasts, glucocorticoids appear to have specific effects on the various *Adamts* proteoglycanase isoforms and genes involved in ADAMTS activation, as dexamethasone treatment was also associated with an increase in *Adamts1*, but not *Adamts5* and -*15*, gene expression, as well as a decrease in *Pcsk6* (but not *Pcsk3*) mRNA transcript abundance. Altogether, these data suggest that glucocorticoids may attenuate the synthesis of a transitional, pericellular matrix, thus facilitating membrane coalescence during fusion in differentiating C2C12 myoblasts [9]. The effects of glucocorticoids on a versican and hyaluronan rich transitional matrix have been described in other biological contexts. Specifically, glucocorticoid induced skin atrophy is associated with reduced proteoglycan [82] and hyaluronan synthesis [44,45]. Glucocorticoids have also been reported to decrease versican expression in cultured rat mesangial cells and airway fibroblasts [43,79].

Myoblast fusion was reduced when differentiating C2C12 myoblasts were treated with versican conditioned media, supporting our hypothesis that excess versican impairs regenerative myogenesis. This decrease in myoblast fusion was ameliorated, but not fully reversed, with dexamethasone. Versikine conditioned media also impaired myoblast fusion, and this impairment was also ameliorated by glucocorticoid treatment. Hyaluronan can bind to versican, and perhaps also versikine, via the G1 N-terminus link module [25], thus contributing to pericellular matrix expansion. We have previously shown that expansion and inadequate processing of a versican–hyaluronan rich pericellular matrix impairs myoblast fusion and myotube formation [9]. This decrease in fusion was interrogated further by quantifying the number of nascent and mature myotubes following versican, versikine and/or dexamethasone treatment. An excess of versican decreased the number of nascent myotubes, and this decrease was ameliorated by dexamethasone treatment. Whereas, an excess of versikine did not compromise nascent myotube formation. Myotube hypertrophy and nuclear accretion occurs via secondary fusion, which involves distinct signalling pathways compared to the formation of nascent myotubes [51]. Excess versican and versikine reduced the formation of mature myotubes. Dexamethasone rescued this impairment and increased the number of mature myotubes formed.

Excess versican or versikine may impair myoblast migration, thus potentially contributing to the observed decrease in myoblast fusion and myotube number. The effects of versican on cell migration are context dependent, as during development, versican has been suggested to deter muscle cell migration and thus contribute to the patterning of the limb skeleton and joints [83]. The underlying mechanism by which versican and versikine regulate cell migration during myogenesis remains to be determined, but may involve CD44 signalling [57,84,85]. Full length versican, through its chondroitin sulphate side chains, can bind to the CD44 receptor directly [84]. Through interactions with the link module on the G1 domain, versican and versikine bind hyaluronan [19], which is also a known ligand for the CD44 receptor [85]. Altogether, these observations suggest a novel mechanism by which excess versican could compromise regenerative myogenesis in muscular dystrophy.

We propose that excess accumulation of versican in dystrophin-deficient muscles compromises regeneration and exacerbates fibrosis. Dadgar et al. [12] have recently confirmed that TGF-β centred signalling networks are key drivers for fibrosis and failed regeneration in muscular dystrophy. TGFβ stimulates V0/V1 versican synthesis in various biological contexts [59,86,87,88]. Furthermore, the interaction between TGF-β and versican is bidirectional, with versican potentiating TGFβ signalling [89] and regulating bioavailability [90]. When dystrophic *mdx* mice were treated with glucocorticoids, these TGF-β centered networks were suppressed and the dystrophic pathology was ameliorated [12]. Therefore, our in vitro observations that glucocorticoids reduce V0/V1 versican (*Vcan*) and *Tgfb1* expression in differentiating myoblasts highlight the relevance of these genes to regenerative myogenesis, especially in the context of dystrophy.

To fully characterise the role of versican in regenerative myogenesis in dystrophic skeletal muscles in vivo studies using genetic and/or pharmacological approaches are needed. The advantage of such in vivo studies is that they will allow the effects of versican on muscle repair and function to be investigated in the presence of an expanded ECM and increased inflammation. This is important, given the emerging role of versican [91,92] and versikine [33] in regulating inflammation in various biological contexts.

## 4. Materials and Methods

### 4.1. Mouse Models

All animal studies were approved by the La Trobe University and Deakin University Animal Ethics Committees, in accordance with the National Health and Medical Research Council (NH&MRC) guidelines, under the ethics numbers of AEC16-08 for La Trobe University (approved: 1 January 2016), and G35-2013 and A79/2011 (approved: 1 January 2015 and 1 January 2012, respectively) for Deakin University. Between 3 and 6 months of age, C57BL/10 (wild type) and *mdx* mice were deeply anaesthetised with sodium pentobarbitone (60 mg/kg) and killed by cardiac excision. TA and diaphragm muscles were collected for immunohistochemical analysis by embedding the blotted tissues in optimal cutting temperature compound (OCT) and freezing in thawing 2-methylbutane cooled in liquid nitrogen. TA and diaphragm muscles were also snap frozen in liquid nitrogen for gene expression analysis.

### 4.2. Skeletal Muscle Immunohistochemistry and Histology

Transverse frozen sections were cut from the mid-belly of the TA or diaphragm muscle strips at a thickness of 8 μm, mounted on slides and stored at −80 °C until analysis. Immunohistochemistry for ADAMTS1 (Origene, TA317919, Rockville, MD, USA), ADAMTS5, ADAMTS15 (Abcam, ab45047, Cambridge, MA, USA), V0/V1 versican (anti-GAGβ; Merck Millipore, AB1033, Bayswater, VIC, Australia) or versikine (anti-DPEAAE neo-epitope; Thermo Fisher Scientific, PA1-1748A, Scoresby, VIC, Australia) were performed as previously described [9,32]. An anti-desmin rabbit polyclonal antibody (Abcam, ab15200, Cambridge, MA, USA) together with an anti-rabbit Alexa Fluor 488 secondary antibody (Thermo Fisher Scientific, R37116,) were used to detect myoblasts and newly regenerated myofibres [93,94]. Representative wild type and *mdx* TA and diaphragm muscle cross-sections were H and E stained for muscle architecture, and wheat germ agglutinin to assess fibrosis [95]. For analysis of V0/V1 versican and versikine immunoreactivity, four non-overlapping representative digital images were captured with a confocal microscope of each muscle section at 600× magnification (Olympus Fluoview FV10i) and analysed for area of immunoreactivity using Image-Pro Plus software (Version 7, Media Cybernetics, Silver Spring, MD, USA).

### 4.3. Cell Culture and Expression Constructs

HEK293T cells were grown in Dulbecco’s Modified Eagle Medium (DMEM; 25 mM glucose) containing 10% fetal bovine serum (FBS) in atmospheric O_2_ and 5% CO_2_ at 37 °C. Cells were transfected using Lipofectamine 2000 (Thermo Fisher Scientific) with constructs encoding the V1 versican construct (kindly provided by Dieter Zimmermann), the bioactive G1-DPEAAE versikine fragment was produced by the insertion of a stop codon in the V1 versican construct after the Glu^441^-Ala^442^ peptide bond cleavage site [32], and empty vector control (pcDNA3.1MycHisA+ (Thermo Fisher Scientific)). Serum-free conditioned medium was collected for use in the myoblast differentiation experiments, as previously described [9], and underwent western blotting for confirmation of V1 versican and versikine protein expression. C2C12 myoblasts, a well characterized in vitro model of myoblast fusion and regenerative myogenesis [96,97], were maintained in growth medium (25 mM glucose DMEM plus 10% FBS) in atmospheric O_2_ and 5% CO_2_ at 37 °C.

### 4.4. Glucocorticoid Treatment of Differentiating C2C12 Cells

To determine the effects of glucocorticoids on myoblast differentiation, cells were seeded at 25,000 cells/cm^2^; in duplicate wells for fusion index determination, and in triplicate wells for gene and protein expression and creatine kinase activity analyses. Following 48 h of proliferation (and at >90% confluence), myoblasts were treated with differentiation medium (25 mM glucose DMEM plus 2% horse serum (HS)) supplemented with 0 nM, 25 nM or 100 nM dexamethasone (Prednisolone F, Sigma-Aldrich, D1756, Castle Hill, NSW, Australia) for 72 h (refreshed every 24 h). The latter is a low glucocorticoid concentration which has been shown to increase myoblast fusion efficiency in vitro [39]. Following this, the cells were harvested for biochemical analyses (see below) or fixed with 4% paraformaldehyde, and stained with Alexa-Fluor 568 phalloidin (1:50; Life Technologies) and DAPI (1:20; Life Technologies) for 20 min.

Fusion index was used as a proxy readout to assess myoblast differentiation efficacy. Nascent myotubes with 3–4 myonuclei and mature myotubes with ≥5 myonuclei were quantified, as previously described [9,50]. For the fusion index, 3 biological replicates (at different passages) in duplicate were performed. For the control cells treated with 0 nM dexamethasone, the % fusion index for each of the three biological replicates ranged from 17–20. For each experimental condition and biological replicate, 8897 ± 222 nuclei were counted to assess fusion index and 270 ± 23 myotubes were classified and counted to assess myotube maturation.

The fusion index data are supported by gene markers of myoblast differentiation and a commercially available creatine kinase enzyme activity assay kit, as per manufacturer’s instructions (ab155901, Abcam) [98]. The cell lysates from the creatine kinase enzyme activity assay were also used for analysis of versican and versikine protein expression.

### 4.5. RNA Extraction, Reverse Transcription, and Quantitative RT-PCR

Cells were harvested in triplicate wells, collected in TRIzol (Sigma-Aldrich) and stored at −80 °C. TA and diaphragm muscles were mechanically homogenised in TRIzol. Upon thawing, RNA was extracted and 1 μg of total RNA was reverse-transcribed using an iScript cDNA synthesis kit (Bio-Rad, Gladesville, NSW, Australia). Quantitative RT-PCR was performed using iQ SYBR Green Supermix (Bio-Rad) and oligonucleotide primers for the murine genes of interest (Table 1). Relative changes in mRNA levels to untreated myotubes were calculated using the Δ*C*_t_ method. Real time data was normalised to cDNA content, as determined using a Quant-iT Oligreen ssDNA reagent kit (Life Technologies).

### 4.6. Versican or Versikine Treatment and C2C12 Myoblast Differentiation

To assess the effects of glucocorticoids on myotube formation in the presence of excess versican or versikine, C2C12 cells were seeded at 20,000 cells/cm^2^ in duplicate wells, 24 h or 48 h later the differentiation medium was added for 4 or 3 days, respectively. Depending on the experimental conditions, the differentiation medium was supplemented with 0 nM or 100 nM dexamethasone and serum-free versican, versikine or empty vector conditioned media (diluted 1:4; refreshed daily). Fusion index and myotube number were determined and analysed as described above. Five biological replicates (at different passages) in duplicate were performed, with a total of 3156 ± 96 nuclei and 110 ± 3 myotubes counted per experimental condition for each biological replicate. For the control cells treated with empty vector conditioned media and 0 nM dexamethasone, the % fusion index for each of three biological replicates ranged from 19–27%. This greater variability in % fusion compared to the dexamethasone dose response experiments reflects the more challenging experimental conditions.

### 4.7. Versican or Versikine Treatment and C2C12 Myoblast Migration and Proliferation

To assess the specific effects of versican and versikine on myoblast migration, a process essential for effective differentiation [50,57], cells were seeded at 8500 cells/cm^2^ in triplicate wells and 72 h later (when 100% confluent) a scratch wound assay was performed [58]. Growth media was supplemented with serum-free versican, versikine or empty vector conditioned media (diluted 1:4) for up to 11 h. To determine migration rate, digital images (three per well) were digitally captured at 0 h, 6 h and 11 h post-wounding. The distance between the edges of the scratches was measured using Adobe Photoshop CS6 (Adobe Systems), with the migration rate calculated in pixels/min.

To assess the effects of versican and versikine on myoblast proliferation, cells were seeded at 10,000 cells/cm^2^ in growth media, supplemented with serum-free versican, versikine or empty vector conditioned media (diluted 1:4; refreshed daily) for 48 h. Myoblast number was assessed using the WST-1 cell proliferation reagent (Roche Life Science, Sydney, NSW, Australia), as per the manufacturer’s directions.

### 4.8. Western Blot

To improve detection by the anti-V0/V1 versican antibody, the GAG side chains were removed. Specifically, 1 μL of chondroitinase ABC (Seikagaku, Tokyo, Japan) was added to the versican conditioned media and to the cell lysates from the creatine kinase enzyme assay for 2 h at 37 °C. The serum free conditioned media containing versikine was subjected to the western blotting as described by Dancevic et al. [30]. The versican conditioned media and C2C12 cell lysates underwent SDS-PAGE using pre-cast Mini-PROTEAN TGX Stain-Free Protein Gels (Bio-Rad), which were transferred onto PVDF membranes. Proteins were visualised by chemiluminescence on a Chemidoc XRS+ (Bio-Rad) and analysed using ImageLab software (Bio-Rad). Versican and versikine protein levels were normalised to total optical density of all protein bands on the TGX Stain-Free Protein Gel. Primary antibodies used were anti-GAGβ (V0/V1 versican) (1:200, Merck Millipore), anti-V0/V1 DPEAAE neo-epitope (versikine) (1:1000, Thermo Fisher Scientific, PA1-1748A), and anti-GAPDH (1:10,000, Merck Millipore, AB1033). Secondary antibodies used were peroxidase AffiniPure goat anti-rabbit IgG (1:5000, Jackson ImmunoResearch Laboratories) and anti-mouse IR680 (1:10,000, Sigma Aldrich).

### 4.9. Statistical Analyses

For the versican gene expression data, 2-way general linear model (GLM) ANOVA was performed with the factors being muscle type (diaphragm versus TA) and strain (C57BL/10 versus *mdx*). For the quantitation of versican and versikine immunoreactivity in TA and diaphragm muscles from *mdx* and wild type mice, independent *t*-tests were used to assess differences in immunoreactivity between C57BL/10 and *mdx* mice for a given muscle type. For the cell culture experiments, independent *t*-tests, 1-way or 2-way GLM ANOVA were performed as appropriate and followed by Tukey’s post-hoc analysis where required. All data are presented as mean ± S.E. and were considered statistically significant when *p <* 0.05.

## Figures and Tables

**Table 1:** List of primer sequences used for quantitative RT-PCR.

Accession Number	Name	Forward Sequence (5′-3′)	Reverse Sequence (5′-3′)
NM_009621.5	*Adamts1*	CCTGTGAAGCCAAAGGCATTG	TGCACACAGACAGAGGTAGAGT
NM_011782.2	*Adamts5*	GCTACTGCACAGGGAAGAGG	GCCAGGACACCTGCATATTT
NM_001329420.1	*Adamts15*	GCTCATCTGCCGAGCCAAT	CAGCCAGCCTTGATGCACTT
NM_007710.2	*Ckm* ^1^	CCGTGTCACCTCTGCTGCTG	TCCTTCATATTGCCTCCCTTCTCC
XM_011250822.2	*Pcsk3* ^2^	CAGCGAGACCTGAATGTGAA	CAGGGTCATAATTGCCTGCT
NM_008216.3	*Has2*	GGGACCTGGTGAGACAGAAG	ATGAGGCAGGGTCAAGCATA
XM_006511645.3	*Hyal2*	AGCCGCAACTTTGTCAGTTT	GAGTCCTCGGGTGTATGTGG
XM_017314318.1	*Myh1* ^3^	GCTCAAAGCCCTGTGTTACC	CATAGACGGCTTTGGCTAGG
NM_001291184.1	*Pcsk6* ^4^	ATTTCCCCAACCTCGTCTCT	AGCTGAGTCCTTGCCACCTA
NM_011577.2	*Tgfb1*	GCCTGAGTGGCTGTCTTTTGA	CACAAGAGCAGTGAGCGCTGAA
NM_001081249.1 (V0)	*V0/V1 Vcan* ^5^	ACCAAGGAGAAGTTCGAGCA	CTTCCCAGGTAGCCAAATCA
NM_019389.2 (V1)			

^1^ Creatine kinase muscle, ^2^ Furin, ^3^ Myosin heavy chain 1 (fast IIx/d isoform), ^4^ Pace4, ^5^ Versican (primers detect the V0 and V1 isoforms).

## Abbreviations

ADAMTS	A disintegrin-like and metalloproteinase domain with thrombospondin-1 repeats
CSPG	Chondroitin sulphate proteoglycan
CK	Creatine kinase
Ckm	Creatine kinase muscle
Dex	Dexamethasone
DMEM	Dulbecco’s modified eagle medium
DMD	Duchenne muscular dystrophy
ECM	Extracellular matrix
EV	Empty vector
FI	Fusion index
FOV	Field of view
GAG	Glycosaminoglycan
GLM	General linear model
GSK-3β	Glycogen synthase kinase-3β
Has	Hyaluronan synthase
HS	Horse serum
Hyal	Hyaluronidase
Myh1	Myosin heavy chain 1
OCT	Optimum cutting temperature
TA	Tibialis anterior
TGF-β	Transforming growth factor-β
Vcan	Versican
